# Supplementary material for: Effect of Different Coffee Brews on Tryptophan Metabolite-Induced Cytotoxicity in HT-29 Human Colon Cancer Cells
Source: Antioxidants (Basel). 2022 Dec 14;11(12):2458. doi: 10.3390/antiox11122458 (PMC9774627; doi:10.3390/antiox11122458)
Supplement: Supplementary file 1 [file antioxidants-11-02458-s001.zip › antioxidants-2052452-SI.pdf]

Supplementary Materials

1. *Coffee preparation*

The espresso coffee brews were prepared from 7 g of Colombian coffee powder, using an Espresso machine (DeLonghi, Icona Vintage, Italy). Then, 25 mL of coffee brew were collected. Americano coffee brew was prepared followed the protocol described by Liu et al., [1]. In short, 200 mL of distilled water was added to the single-shot espresso and thoroughly mixed. Instant coffee brews were prepared by pouring 200 mL of boiling distilled water over 6 g of instant coffee powder and stirred until dissolved as indicated by the manufacturer. Each coffee brew sample was obtained at least ten times, pooled, and stored at -18 °C until analysis.

2. *In Vitro Simulated Gastrointestinal Digestion*

Coffee brews samples were brought under successive oral, gastric and intestinal in vitro digestion, following a harmonized procedure recently created by the COST action INFOGEST network [2]. Simulated digestion fluids, namely gastric fluid (SGF), salivary fluid (SSF) and intestinal fluid (SIF) were built considering a previously described procedure [3] and showed in Table S1.

Briefly, 3.5 mL of SSF (T=37 °C) were added to 5 mL of sample and mixed. Next, 25 μL of 0.3 M calcium chloride, 0.5 mL of α-amylase solution (75 U/mL), and 975 μL of water were added and mixed. A solution 1 M HCl was added to reduce the pH of the solution to 7, and the mixture was incubated at 37 °C for 2 min in an orbital shaker bath at 100x *g*.

Then, for simulating gastric condition, 1.6 mL pepsin solution (2000 U/mL), 7.5 mL SGF, 695 μL of water and 5 μL 0.3 M calcium chloride were added and thoroughly mixed. Next, HCl 1 M was used to decrease the pH of the solution to 3, and the mixture was incubated for 120 min at 37 °C in an orbital shaker bath at 50 *g*.

Afterward, to recreate the intestinal stage, 11 mL SIF, 5 mL pancreatin solution (100 U/mL of trypsin activity), 2.5 mL bile salt solution (65 mg/mL), 1.3 mL of water and 40 μL of 0.3 M calcium chloride were added. After that, the solution was thoroughly mixed, and 1 M NaOH was added to increase the pH of the mixture to 7. The solution was incubated at 37 °C for 120 min in an orbital shaker bath at 100x *g*, and then, centrifuged for 10 min at 37 °C at 4900x *g*.

Finally, the remaining pellet was subjected to the previously described procedure [3]. First, 5 mL of 1 mg/mL Pronase E solution were added, and pH was readjusted to 8 using NaOH 1M, simulating the action of the intestinal microbiota. The mixture was then incubated at 37 °C for 60 min in an orbital shaker bath at 100x *g*. Next, the mixture was treated with 150 μL of Viscozyme L. The pH was readjusted to 4 and incubated at 37 °C for 16 h and centrifuged at 4900x *g* for 10 min at 24 °C.

References

1. Liu, Z.-S.; Chen, P.-W.; Wang, J.-Y.; Kuo, T.-C., Assessment of Cellular Mutagenicity of Americano Coffees from Popular Coffee Chains. *Journal of food protection* **2017,** 80, (9), 1489-1495.
2. Minekus, M.; Alminger, M.; Alvito, P.; Ballance, S.; Bohn, T.; Bourlieu, C.; Carriere, F.; Boutrou, R.; Corredig, M.; Dupont, D., A standardised static in vitro digestion method suitable for food–an international consensus. *Food function* **2014,** 5, (6), 1113-1124.
3. Izzo, L.; Rodríguez-Carrasco, Y.; Pacifico, S.; Castaldo, L.; Narváez, A.; Ritieni, A., Colon Bioaccessibility under In Vitro Gastrointestinal Digestion of a Red Cabbage Extract Chemically Profiled through UHPLC-Q-Orbitrap HRMS. *Antioxidants* **2020,** 9, (10), 955.
